# 

**Published:** 1859-03

**Authors:** 


SESSION 1858-'59.

ELISHA TOWNSEND, D. D. S.,

EMERITUS PROF. OF OPERATIVE DENTISTRY.

### FACULTY.

C. N. PIERCE, D. D. S.,

PROF. OF DENTAL PHYSIOLOGY AND OPERATIVE DENTISTRY.

T. L. BUCKINGHAM, D. D. S.,

PROF. OF CHEMISTRY AND METALLURGY.

J. L. SUESSEROTT, D. D. S.,

PROF. OF PRINCIPLES OF DENTAL SURGERY AND THERAPEUTICS.

J. H. McQUILLEN, D. D. S.,

PROF. OF ANATOMY AND PHYSIOLOGY.

WILLIAM CALVERT, D. D. S.,

PROF. OF MECHANICAL DENTISTRY.

D. H. GOODWILLIE, D. D. S.,

DEMONSTRATOR OF OPERATIVE DENTISTRY.

T. W. WALKER, D. D. S.,

DEMONSTRATOR OF MECHANICAL DENTISTRY.

The regular Course will commence on the first Monday of November, and continue until the 1st of March ensuing.

During October, the Laboratory will be open, and a Clinical Lecture delivered every Saturday by one of the Professors, at 3 o'clock, P. M.

The most ample facilities are furnished for a thorough course of practical instruction.

Tickets for the Course, Demonstrators' Tickets included, $100; Matriculation Fee $5; Diploma Fee $30.

For further information, address

T. L. BUCKINGHAM, DEAN,
No. 243 North Ninth Street.

### JOHN B. DUNLEVY,

### GOLD AND TIN FOIL, GOLD PLATE &c.,

**No. 126 Wood Street, Pittsburgh, Pa.**

☞Orders promptly and faithfully attended to

---

### CHARLES ABBEY & SONS,

MANUFACTURERS OF

### DENTISTS' FINE GOLD,

### AND TIN FOIL,

**Nos. 228 and 230 Pear Street,**

PHILADELPHIA.

---

### BALTIMORE COLLEGE OF DENTAL SURGERY.

**NINETEENTH ANNUAL SESSION 1858-59.**

### FACULTY.

CHAPIN A. HARRIS, D. D. S., M. D.
*Principles of Dental Science and Practice of Dental Surgery.*

THOMAS E. BOND, M. D.
*Therapeutics and Materia Medica.*

PHILIP H. AUSTEN, D. D. S., M. D.
*Mechanical Dentistry.*

REGINALD N. WRIGHT, M. D.
*Chemistry and Metallurgy.*

A. SNOWDEN PIGGOT, M. D.
*Anatomy and Physiology.*

CHRISTOPHER JOHNSTON, M. D.
*Microscopical and Comparative Anatomy of the teeth.*

FERDINAND J. S. GORGAS, D. D. S.,
*Demonstrator of Mechanical Dentistry.*

SAMUEL T. CHURCH, M. D., D. D. S.
*Demonstrator of Operative Dentistry.*

The Nineteenth Annual Session will commence on the 4th day of October, and close on the 1st of March.

The regular course of LECTURES will commence on the 1st of November.

The entire day during October, and the morning of each day during the rest of the session, will be devoted to Practical Dentistry and Anatomical Dissections.

The Infirmary has a large and rapidly increasing supply of patients, affording to the student unsurpassed facilities for the acquirement of a PRACTICAL knowledge of every Operation in Surgical Medical and Mechanical Dentistry.

Professors' Tickets, $100 : Demonstrators' Tickets, $20 ; Matriculation Fee, $5 ; Diploma Fee, $30. Dissection Ticket, (optional), $10.

For further information, address **P. H. AUSTEN**, DEAN OF THE FACULTY,
79 North Charles Street, Baltimore.

